# A systematic overview of single-cell transcriptomics databases, their use cases, and limitations

**DOI:** 10.3389/fbinf.2024.1417428

**Published:** 2024-07-08

**Authors:** Mahnoor N. Gondal, Saad Ur Rehman Shah, Arul M. Chinnaiyan, Marcin Cieslik

**Affiliations:** ^1^ Department of Computational Medicine and Bioinformatics, University of Michigan, Ann Arbor, MI, United States; ^2^ Michigan Center for Translational Pathology, University of Michigan, Ann Arbor, MI, United States; ^3^ Gies College of Business, University of Illinois Business College, Champaign, MI, United States; ^4^ Department of Pathology, University of Michigan, Ann Arbor, MI, United States; ^5^ Department of Urology, University of Michigan, Ann Arbor, MI, United States; ^6^ Howard Hughes Medical Institute, Ann Arbor, MI, United States; ^7^ University of Michigan Rogel Cancer Center, Ann Arbor, MI, United States

**Keywords:** single-cell RNA-seq, single-cell databases, single-cell atlases, single-cell data analysis, web-based platforms, cell heterogeneity, single-cell data integration, computational methods

## Abstract

Rapid advancements in high-throughput single-cell RNA-seq (scRNA-seq) technologies and experimental protocols have led to the generation of vast amounts of transcriptomic data that populates several online databases and repositories. Here, we systematically examined large-scale scRNA-seq databases, categorizing them based on their scope and purpose such as general, tissue-specific databases, disease-specific databases, cancer-focused databases, and cell type-focused databases. Next, we discuss the technical and methodological challenges associated with curating large-scale scRNA-seq databases, along with current computational solutions. We argue that understanding scRNA-seq databases, including their limitations and assumptions, is crucial for effectively utilizing this data to make robust discoveries and identify novel biological insights. Such platforms can help bridge the gap between computational and wet lab scientists through user-friendly web-based interfaces needed for democratizing access to single-cell data. These platforms would facilitate interdisciplinary research, enabling researchers from various disciplines to collaborate effectively. This review underscores the importance of leveraging computational approaches to unravel the complexities of single-cell data and offers a promising direction for future research in the field.

## 1 Introduction

The first commercially available single-cell platform emerged in 2014 (Wu et al., 2018). Over the past decade, single-cell sequencing technologies have rapidly advanced, becoming faster and more cost-effective. Today, there are over 10 different commercially available platforms for high-throughput single-cell data collection (Valihrach et al., 2018; Mereu et al., 2020). This advancement has fueled remarkable growth in the field of single-cell RNA sequencing (scRNA-seq) research, with nearly 2000 studies published to date (Svensson et al., 2020), populating numerous databases and repositories (Regev et al., 2017; Abugessaisa et al., 2018; Franzén et al., 2019; Wang et al., 2019; Papatheodorou et al., 2020). These studies have provided valuable insights into various biological processes, including development (Han et al., 2020), disease initiation and progression (Strzelecka et al., 2018), immune response (Chen et al., 2023), and identification of rare cell types (Lee et al., 2019; Salcher et al., 2022). Alongside the generation of large-scale single-cell data, we also observe a sharp rise in scRNA-seq analysis tools, expected to reach 3,000 by the end of 2025 (Zappia and Theis, 2021; Davis, 2019).

Previous reviews and benchmarking analyses have extensively covered various aspects of scRNA-seq analysis such as quality control (Lähnemann et al., 2020), normalization (Hafemeister and Satija, 2019; Sina Booeshaghi et al., 2022), integration (Tran et al., 2020; Luecken et al., 2022), and cell type annotation (Abdelaal et al., 2019). However, the complexity of large-scale data necessitates a comprehensive evaluation of available scRNA-seq databases and repositories. This evaluation is crucial to understand concepts like integration in the context of large-scale databasing. Understanding the scope and limitations of these databases is crucial for storing, analyzing, and interpreting single-cell data directly from these repositories. In this review, we systematically address the limitations and common assumptions of existing scRNA-seq databases. We discuss the utility of these databases to meet the specific needs of researchers studying different biological systems and processes.

## 2 Landscape of single-cell transcriptomics databases

**FIGURE 1:**
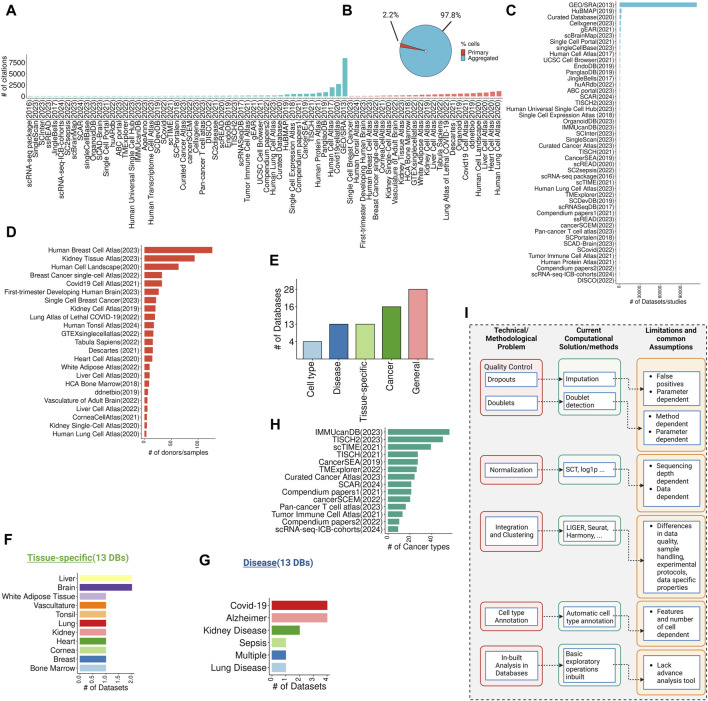
Overview of single-cell databases, their technical/methodological issues, current solutions, and common assumptions. **(A)** Overview of citations gathered from single-cell data repositories from primary or aggregated studies (data collected on March 31st). **(B)** The pie chart showing the total cells in the primary and aggregated data source **(C)** Highlights the number of datasets or studies published per database in the primary data source category. **(D)** Shows the number of donors/samples in the aggregated data source category. **(E)** A bar chart highlighting the total percentage of databases in general, tissue-specific, disease-specific, cancer-focused, and cell type-focused databases. **(F,G)** Exhibits the number of databases/studies for tissue-specific and disease-focused databases. **(H)** Shows the number of cancer types per cancer-focused databases. **(I)** Technical and methodological issues with databases, current computational and methods-based solutions, and their common assumptions and limitations.

The rapid expansion of single-cell RNA-seq (scRNA-seq) studies has led to the development of numerous databases and repositories for storing, retrieving, and interpreting single-cell data (Regev et al., 2017; Abugessaisa et al., 2018; Franzén et al., 2019; Wang et al., 2019; Papatheodorou et al., 2020). These databases provide a resource for single-cell transcriptomic data that can be used to build computational models to investigate various biological processes. The data in scRNA-seq databases or atlases can come from either a “primary” source, that exclusively hosts data generated by the study itself and is not shared or aggregated with data from other studies, or an “aggregated” source, where data was collected and curated from multiple studies (Figures 1A–D). Single-cell databases can be further categorized into general (non-specific or broad category of databases), tissue-specific databases, disease-specific databases, cancer-focused databases, and cell type-focused databases (Figure 1E). We have curated a comprehensive list of scRNA-seq databases, accessible here (https://github.com/mctp/single-cell-Databases/tree/main, Supplementary Table S1). This list includes database names, years of establishment, PubMed IDs, citation counts (as of March 31st), URLs, web interface availability, number of datasets/studies, cell counts, primary vs. aggregated distinction, specific groups, tissue types, data types, file types, normalization methods (if mentioned), data locations, and types of web interfaces.

### 2.1 General single-cell databases

The establishment of the Human Cell Atlas (HCA) (Regev et al., 2017) in 2017 marked a significant effort to collect and integrate large-scale single-cell data into a comprehensive reference atlas for all human cells. HCA’s open-access resource forms one of the largest public databases for integrated single-cell data from large-scale sequencing projects comprising over 437 projects and 58.5 million cells across 18 tissues. Single-cell atlases offer high-resolution views of cellular composition in organs, leading to groundbreaking discoveries of rare cell types, developmental processes, and cell states associated with various disease processes (Argelaguet et al., 2021; Rood et al., 2022; Badia-I-Mompel et al., 2023).

Other general databases include Single Cell Portal (SCP) (Tarhan et al., 2023) and CZ CellxGene Discover (CZI Single-Cell Biology Program et al., 2023) which are more flexible and are ideal for retrieving single-cell data focusing on a particular dataset of interest, focusing on unique features and variations within the datasets. The SCP and CZ CellxGene, developed by the Chan Zuckerberg Initiative (CZI) and Broad Institute, respectively, provide web-based interfaces for data exploration and analysis. CZ CellxGene hosts more than 1,284 datasets while SCP constitutes 654 datasets. They offer interactive visualization tools for exploring gene expression patterns, cell clusters, and cell type annotations in scRNA-seq data. Both platforms support the sharing of scRNA-seq datasets, allowing researchers to collaborate and access public datasets. Using the CZ CellxGene platform, users can also download the raw count data as an RDS file containing a Seurat object or an h5ad file with an AnnData object to perform their analysis. Similarly, SCP data can be downloaded as individual metadata files, raw count expressions, and normalized expression data from the website directly, however, the availability of raw or normalized data is subjective to each study in SCP. Another interesting example of a comprehensive database is Tabula Sapiens (Sapiens Consortium* et al., 2022) which houses primary data from 15 individuals across 24 tissues. This database enables the evaluation of gene expression in normal or baseline cell states, providing a valuable resource for developing gene regulation networks and trajectories (Gondal et al., 2024a). It offers a unique opportunity to study cell type-specific expression changes. The data is easily accessible through web platforms and can also be explored using tools like CZ CellxGene.

### 2.2 Tissue-specific single-cell databases

While cross-tissue general databases have their advantages, such as the ability to compare gene expression across different tissues and identify commonalities or differences. However, tissue-specific databases offer a more focused and detailed view of a particular tissue’s biology, making them valuable resources for researchers studying specific tissues. These databases can also provide in-depth insights into specialized functions, cell types, and tissue-specific immunity (Elmentaite et al., 2022). Towards this goal, one of the HCA’s sub-projects aims to develop tissue-specific reference atlases that serve as consensus representations of specific organs across multiple projects (Figure 1F). These atlases provide a standardized reference for specific tissues for comparing different datasets, facilitating cross-study comparisons and meta-analyses. An example of a tissue-specific single-cell database established by the HCA is the Human Lung Cell Atlas (HLCA) (Sikkema et al., 2023) which integrates 49 lung datasets encompassing 2.4 million cells from 486 individuals. The HLCA database development involved four main steps: data curation, integration method selection, cell type annotation, and data usage. Despite the benefits associated with large-scale tissue-specific single-cell databases, it is important to note that integrating data from diverse datasets, labs, and technologies presents challenges due to differences in data quality, sample handling, and experimental protocols. Moreover, ensuring consistency and standardization across datasets is crucial for meaningful comparisons, but achieving this in practice can be complex, particularly with the wide variety of cell types and states. For the HLCA project, the team primarily relied on harmonized manual annotation, integration benchmarking (Luecken et al., 2022), and insights from experts in the field, which can be subjective and may lack reproducibility.

### 2.3 Disease-focused single-cell databases

These general databases are not designed to systematically gather data on gene expression specificity in different diseases. Given the diverse and heterogeneous nature of human diseases, which manifest unique gene expression profiles, there is a critical need for databases focused on disease-specific exploration (Figure 1G).

SC2disease (Zhao et al., 2021) is a manually curated scRNA-seq database that addresses this need, cataloging cell type-specific genes associated with 25 diverse diseases, including Huntington’s disease, multiple sclerosis, and Alzheimer’s disease. While SC2disease represents a pioneering effort in disease-specific gene expression profiling, there is also a growing need for more specific databases dedicated to the disease of interest. To address this, databases like SC2sepsis (Li et al., 2022), ssREAD (Wang C. et al., 2023), and SCovid (Qi et al., 2022) have emerged, focusing on individual diseases such as sepsis, Alzheimer’s, and COVID-19, respectively. These databases aim to provide a more granular and disease-specific view of gene expression patterns, enhancing our understanding of disease mechanisms and potential therapeutic targets.

### 2.4 Cancer-focused single-cell databases

Cancer is a complex disease characterized by its highly heterogeneous and multifactorial nature (Gondal and Chaudhary, 2021). Traditional approaches to studying cancer, such as bulk RNA sequencing constitute a mixture of the cellular composition in tumors and often fail to accurately capture cancer cell-specific gene expression (Ding et al., 2020; Huang et al., 2023). scRNA-seq technologies offer unprecedented insights into tumor heterogeneity, evolution, and responses to therapy (Wang et al., 2020; Zeng et al., 2022; Gondal et al., 2024b). As a result, numerous databases hosting cancer-focused scRNA-seq data have emerged (Figure 1H).

One such example is CancerSEA (Yuan et al., 2019) which was launched in 2019 as a resource utilizing single-cell data from cancer datasets to decode the functional states of cancer cells, these states included stemness, invasion, metastasis, proliferation, epithelial-to-mesenchymal transition (EMT), angiogenesis, apoptosis, cell cycle, differentiation, DNA damage, DNA repair, hypoxia, inflammation, and quiescence. In a salient study, Dohmen et al. (Dohmen et al., 2022) utilized CancerSEA’s functional states to validate gene sets derived from their machine-learning model, while Zhao et al. (Zhao et al., 2023) demonstrated the necessity of NF-KB for initiating oncogenesis using CancerSEA’s functional states. Several other studies (Lan et al., 2020; Deng et al., 2022; Tang et al., 2022; Wang L. et al., 2023) have leveraged CancerSEA to correlate their gene or gene set findings with cancer single-cell data, showcasing the utility of this resource.

However, CancerSEA has limitations, including hosting only 93,475 malignant cells and an inability to study interactions between stromal or immune cells and cancer cells. It also lacks a user-friendly web interface to support data exploration and visualization. In an attempt to overcome these challenges, TISCH was originally developed in 2021 (Sun et al., 2021), with version two released in 2023 (Han et al., 2023). TISCH2 curates cancer datasets with both malignant and non-malignant cell types, currently hosting 190 datasets, encompassing 50 cancer types, and spanning 6 million cells. To illustrate the utility of TISCH in computational models, Xu et al. (2023) employed TISCH’s data to analyze the correlation between FOXM1 and immune cells. Similarly, Zhang et al. (2023) employed TISCH to evaluate m7G regulators expression in osteosarcoma scRNA-seq data. As such, numerous studies have utilized TISCH to evaluate the expression of genes of interest across cancer datasets (Zhao et al., 2022; Liu et al., 2023a; Benedetti et al., 2023; Liu et al., 2023b; Liu Y. et al., 2023; Zhang et al., 2023). Although TISCH provides a valuable resource to the cancer research community, it is important to be aware of the assumptions and limitations of TISCH data. While many studies aim to understand gene expression in malignant cells, TISCH also contains treatment data from immune checkpoint blockade (ICB), chemotherapy, and targeted therapy. Therefore, it is important to ensure that the results are not confounded, as gene expression varies after treatments and can yield diverse results (Liu et al., 2024). Additionally, TISCH includes data from multiple stages of cancer, such as primary tumors or metastatic sites. Therefore, users need to carefully extract only relevant information when employing TISCH data. TISCH employs an automatic cell-type annotation method, which may lead to a lack of consensus with the original dataset’s manual annotation. Importantly, all downloaded datasets in TISCH are in fixed expression matrices (Zeng et al., 2022), and users cannot download the raw count data. Therefore, any attempts to further integrate or normalize the data might result in technical variation rather than biological results. These limitations could potentially introduce bias into analyses or hinder comparability across different databases.

### 2.5 Cell-type-focused single-cell databases

To better understand the intricacies of cell biology, dedicated resources focused on cell-type profiling of single cells have emerged. JingleBells (Ner-Gaon et al., 2017), introduced in 2017, represented an advancement in this direction by providing a comprehensive immune cell resource. JingleBells facilitates the study of immune cell involvement in various diseases, including cancer, and infectious diseases, providing valuable insights into disease mechanisms and potential therapeutic targets. However, JingleBells lacks an interactive web interface and only allows for BAM file download which means analyzing and interpreting single-cell data from JingleBells requires specialized computational tools and expertise, limiting accessibility to researchers with specific skills. In comparison, the human Antigen Receptor database (huARdb) (Wu et al., 2022), published originally in 2022, is a comprehensive human single-cell immune profiling database, housing 444,794 high-confidence T or B cells (hcT/B cells) with complete TCR/BCR sequences and transcriptomes sourced from 215 datasets. To enhance user experience, the authors have created a user-friendly web interface that offers interactive visualization modules, enabling biologists to analyze transcriptome and TCR/BCR features at the single-cell level with ease. Fan et al. (2023a) utilized huARdb by analyzing ulcerative colitis (UC) patients’ immune cells derived from huARdb. Similarly, they also employed huARdb to investigate the healthy and UC composition of peripheral blood immune cells and colonic cells (Fan et al., 2023b). Additional cell-type-focused single-cell databases include EndoDB (Khan et al., 2019) which hosts endothelial cells transcriptomics data from 360 datasets and ABC portal (Gao et al., 2023), a database for blood cells across 198 datasets, allowing for a blood cell-type-specific exploration.

## 3 Challenges associated with the utilization of large-scale single-cell databases and their examples from current literature

While the scRNA-seq field is progressing towards the improvement and development of large-scale single-cell databases, their application in research comes with certain caveats and despite their vastness, they must be used judiciously (Figure 1I). Some of the key considerations and limitations include:

### 3.1 Data quality

Ensuring data quality in scRNA-seq is critical for accurate interpretation and analysis. A fundamental assumption of droplet-based scRNA-seq is that each droplet, where molecular tagging and reverse transcription occur, contains messenger RNA (mRNA) from a single cell. However, in practice, this assumption is often violated, leading to potential distortions in the interpretation of scRNA-seq data. Common examples include doublets which are real cells however they contain multiple cells and dropouts where the expression of one gene was not detected in one cell. This becomes a major issue because large-scale databases such as the Human Cell Atlas (HCA) rely heavily on the accuracy and cellular specificity of transcriptional readouts generated by scRNA-seq.

#### 3.1.1 Examples from current literature and benchmarking studies

##### 3.1.1.1 Dropouts

To overcome the issue of dropouts, numerous single-cell imputation methods have been developed. However, imputation affects downstream results and some of these methods may introduce false correlations. For example, Breda et al. (2021)’s comparison of MAGIC (van Dijk et al., 2018) results with Sanity (SAmpling-Noise-corrected Inference of Transcription activitY), elicited that MAGIC introduced strong positive correlations where no or low correlation was expected. A comparative study by Zhang and Zhang (2020) highlighted that the number of cells and method parameters also affected imputation results and some methods preferred similar cells while imputing. Therefore, imputation results can be variable, and downstream analysis will be affected by imputation.

##### 3.1.1.2 Doublets

Doublets are major confounders in scRNA-seq data analysis. However, there are computational methods that exist to detect doublets in single-cell data. A benchmarking study (Xi and Li, 2021) compared nine doublet detection methods, revealing that there is still room for improvement in detection accuracy. Generally, these methods performed better on datasets with higher doublet rates, larger sequencing depths, more cell types, or greater heterogeneity between cell types. However, the removal of doublets by these methods led to improvements in various downstream analyses. It enhanced the identification of Differentially Expressed (DE) genes, reduced the presence of spurious cell clusters, and improved the inference of cell trajectories. However, the extent of improvement varied across different methods, highlighting the need for further refinement and development in this area.

### 3.2 Normalization

Normalization is another critical aspect of scRNA-seq data analysis and can be a complex problem when dealing with multiple datasets. Specifically, variability in experimental protocols and data processing methods can pose challenges in data normalization, affecting the comparability of results across datasets in a database. Differences in normalization approaches can lead to discrepancies in gene expression profiles, making it difficult to draw meaningful conclusions from the downstream analyses.

#### 3.2.1 Examples from current literature and benchmarking studies

There are several methods to perform single-cell data normalization such as SCT transformation, and log1p normalization (Hafemeister and Satija, 2019). The choice of the method, however, is dependent on various features of the data including sequencing depth as both lowly and highly abundance genes are confounded by sequencing depth (Hafemeister and Satija, 2019). Sina Booeshaghi et al. (2022) demonstrated that the assumptions implied in the choice of normalization methods will affect downstream analysis in determining whether the variation is technical or biological. In a salient example, TISCH2 (Han et al., 2023) database hosts single-cell gene expression matrices for each dataset. In our analysis of TISCH2 data, this matrix is already normalized and integrated, users incorporating this data in their research need to be aware of this normalization to make accurate assessments of data and not re-normalize or merge it directly with other datasets which might result in substandard results. Therefore, in our opinion, when using datasets directly from single-cell databases it is necessary to be aware of the pre-processing steps and how they affect downstream results to ensure accurate analysis and interpretation.

### 3.3 Integration and batch effects removal

The integration and batch effect removal of scRNA-seq data from diverse datasets, labs, and technologies can be complex (Stuart et al., 2019). Variations in data formats, processing pipelines, and batch effects can affect the robustness and reliability of integrated analyses, potentially masking true biological signals. Methods for integrating heterogeneous datasets are continually evolving, with efforts focused on minimizing batch effects and preserving biological variability. There are more than 50 integration methods published to date (Zappia et al., 2018; Luecken et al., 2022).

#### 3.3.1 Examples from current literature and benchmarking studies

Large databases host numerous datasets from multiple studies, however, it is also important to be aware of the properties associated with each study during integration. For example, Salcher et al. (2022) established a large non-small cell lung cancer (NSCLC) atlas comprising 29 datasets spanning 1,283,972 single cells from 556 samples. Although this effort resulted in the in-depth characterization of a neutrophil subpopulation, however, according to our re-analysis of this data, among the 29 datasets, Maynard et al. (2020)’s NSCLC samples were also incorporated which were not treatment-naive. This can be a potential confounder in downstream analysis. Therefore, it is the user’s responsibility to be aware of this data-specific property and to use atlases and databases with care to derive robust biological insights. Similarly, several attempts have been made to benchmark integration methods for single-cell data (Tran et al., 2020; Luecken et al., 2022). While Tran et al. (2020) showed that LIGER (Liu et al., 2020), Seurat 3 (Satija et al., 2015), and Harmony (Korsunsky et al., 2019) performed the best among 11 other methods, Luecken et al. (2022) revealed that LIGER and Seurat v3 favor the removal of batch effect over the conservation of biological variation. This highlights the importance of considering the dataset and the specific research question when selecting an integration method. Selecting the right method is crucial as it directly impacts the biological insights that can be generated from the integrated data.

### 3.4 Cell-type annotation

Accurate annotation of cell types in scRNA-seq databases is crucial for interpreting results accurately. Harmonizing cell type annotations across different datasets is essential for facilitating cross-study comparisons and meta-analysis. While automatic cell-type annotation methods are convenient, they may lack consensus with manual annotations from original datasets. This can introduce ambiguity in cell-type assignments and lead to misinterpretation of results.

#### 3.4.1 Examples from current literature and benchmarking studies

In our recent re-analysis of Tabula Sapiens data (Sapiens et al., 2022), we observed that 10% of the heart cells were mislabelled as hepatocytes in the study’s original metadata. This is biologically incorrect since hepatocytes cannot be in the heart, these are liver epithelial cells (Gong et al., 2022). One potential reason for this mislabelling can be that Tabula Sapiens data was annotated using an automatic cell-type annotation tool, another reason could be sample mishandling. Therefore, diligent manual intervention for cell type annotation needs to be practiced to ensure accurate and robust results. Additionally, Abdelaal et al. (2019) carried out a performance comparison analysis between 22 automatic cell-type identification methods in single-cell data. Although the authors did not state a preference they noted that the results can vary depending on input features and the number of cells which means that they cannot be solely relied on, there will be some manual intervention for accurate cell type annotation.

### 3.5 “Zero-code” single-cell analysis platforms

Single-cell data plays a crucial role in validating and enhancing the accuracy of wet lab results and hypothesis-driven publications (Bao et al., 2023). To facilitate easy access and analysis of this data, many databases provide built-in tools that allow researchers without computational expertise to explore existing datasets and assess their hypotheses using basic operations like exploring gene expressions, isolating cell subsets for individual analysis, and identifying clusters within the data. However, for more complex analyses that require significant computational resources, these tools are often not available directly on the database platforms.

To address this challenge, several web-based platforms have been created to enable online analysis of scRNA-seq data. Among them, the Automated Single-cell Analysis Pipeline (ASAP) (Gardeux et al., 2017) was published in 2017 and provided basic processing analysis of scRNA-seq data post-alignment from filtering to cell type annotation and functional gene set enrichment. Later, this web-based pipeline improved in 2020 (David et al., 2020) with more scalable options. However, ASAP did not include advanced scRNA-seq analysis tools such as regulon activity assessment. To address this issue, ICARUS_v2 (Jiang et al., 2023a) was launched which also incorporates the Drug-Gene Interaction database to facilitate drug discovery. This platform was improved and ICARUS_v3 (Jiang et al., 2023b) was published for zero-code single-cell analysis. ICARUS_v3 employs a geometric cell sketching method to subsample representative cells from the dataset to store in memory. This enables advanced scRNA-seq analysis through a user-friendly web interface. ICARUS_v3 can seamlessly integrate with output files from databases like Single Cell Portal (SCP) (Tarhan et al., 2023) and CZ CellxGene Discover (CZI Single-Cell Biology Program et al., 2023), eliminating the need for coding expertise. Users can leverage this platform to conduct a wide range of analyses, including differential expression analysis, gene regulatory network construction, trajectory analysis, and cell-cell communication inference. While such tools facilitate online analysis of scRNA-seq data, offering user-friendly interfaces and automated workflows. However, these platforms come with limitations and assumptions. They often assume users have high-quality, pre-processed data and a stable internet connection, which may not always be the case. The platforms also impose constraints on data size and complexity due to server limitations, potentially limiting the depth of analysis for large or intricate datasets. Additionally, the algorithms and default parameters embedded in these platforms may not be optimal for all types of scRNA-seq data, leading to less tailored analyses compared to custom pipelines. Despite these limitations, such platforms provide valuable accessibility and convenience for many researchers.

## 4 Platforms for hosting and visualizing large-scale single-cell data

As the volume of single-cell data continues to grow, scalability becomes a significant concern. Developing methods and infrastructure that can handle the increasing complexity and size of single-cell datasets is crucial for future research. Towards this aim, for easy, fast, and customizable exploration of single-cell data for public use, numerous user-driven platforms have emerged (Rue-Albrecht et al., 2018; Feng et al., 2019).

One such platform, the Interactive SummarizedExperiment Explorer (iSEE) (Rue-Albrecht et al., 2018), launched in 2018, enables users to host their SummarizedExperiment data. Researchers such as Graf et al. (2019) and Newton et al. (2022) have employed iSEE to visualize their single-cell data, demonstrating its utility in data exploration. Similarly, the Single Cell Explorer (Feng et al., 2019) allows users to input loom and Seurat objects, making the data more accessible.

ShinyCell (Ouyang et al., 2021) is another example of a platform offering web-based interfaces for exploring and analyzing data. These interfaces can be customized for maximum usability and can be uploaded to online platforms to broaden access to published data. ShinyCell supports various common single-cell data formats, including SingleCellExperiment, h5ad, loom, and Seurat objects, as inputs. In a salient example, Ma et al. (2023) used ShinyCell to host their pan-cancer single-cell data, showcasing its versatility and effectiveness in data dissemination. Likewise, Curras-Alonso et al. (2023) developed their web application using ShinyCell, highlighting its widespread adoption in the research community. By providing easy-to-use tools for data analysis, these platforms help democratize access to single-cell data and facilitate collaboration between researchers from different disciplines.

## 5 Discussion

The rapid expansion of single-cell RNA-seq (scRNA-seq) studies has ushered in a plethora of databases and repositories dedicated to storing, retrieving, and interpreting single-cell data. These databases provide a wealth of single-cell transcriptomic data that can be used to build computational models to understand various biological processes. However, challenges such as data quality, normalization, integration, and annotation can affect the reliability and comparability of results across different datasets and studies.

While the existing databases are valuable for basic scRNA-seq analysis, they cannot often perform advanced analyses such as regulon activity assessment, pseudobulking, and differential gene expression analysis. Users still need to possess programming skills and be familiar with using a command-line interface to conduct customized analysis. Furthermore, many wet labs may not have the necessary resources to manage high-performance computing clusters. To address this gap and enable wet-lab researchers to conduct advanced scRNA-seq analysis, platforms like ICARUS_v3 (Jiang et al., 2023b) offer web-based analysis tools. These platforms provide an accessible way for researchers to explore and analyze single-cell data, bridging the gap between wet lab experimentation and bioinformatics analysis.

Taken together, in this mini-review, we address the utility and applicability of large-scale scRNA-seq databases. We address some of the challenges and common assumptions that need to be considered when using these databases for hypothesis-driven studies, highlighting platforms for hosting customized scRNA-seq data for community usage. While challenges remain, the development of user-friendly platforms is narrowing the gap between wet-lab experimentation and bioinformatics analysis, ultimately advancing our understanding of cellular processes at a single-cell level.
